# Serum Matrix Metalloproteinase-3 in Comparison with Acute Phase Proteins as a Marker of Disease Activity and Radiographic Damage in Early Rheumatoid Arthritis

**DOI:** 10.1155/2013/183653

**Published:** 2013-04-07

**Authors:** Mahmood M. T. M. Ally, Bridget Hodkinson, Pieter W. A. Meyer, Eustasius Musenge, Mohammed Tikly, Ronald Anderson

**Affiliations:** ^1^Department of Internal Medicine, Faculty of Health Sciences, University of Pretoria, Private Bag X663, Pretoria 0001, South Africa; ^2^Division of Rheumatology, Department of Medicine, Chris Hani Baragwanath Hospital, University of the Witwatersrand, Johannesburg, South Africa; ^3^Medical Research Council Unit for Inflammation and Immunity, Department of Immunology, Faculty of Health Sciences, University of Pretoria and Tshwane Academic Division of the National Health Laboratory Service, Pretoria, South Africa; ^4^Biostatistics and Epidemiology Division, School of Public Health, Faculty of Health Sciences, University of the Witwatersrand, Johannesburg, South Africa

## Abstract

Matrix metalloproteinase-3 (MMP-3) is involved in the immunopathogenesis of rheumatoid arthritis (RA), but little is known about its relationship to genetic susceptibility and biomarkers of disease activity, especially acute phase reactants in early RA. MMP-3 was measured by ELISA in serum samples of 128 disease-modifying, drug-naïve patients and analysed in relation to shared epitope genotype, a range of circulating chemokines/cytokines, acute phase reactants, autoantibodies, cartilage oligomeric protein (COMP), and the simplified disease activity index (SDAI). MMP-3 was elevated >1.86 ng/ml in 56.25% of patients (*P* < 0.0001), correlated with several biomarkers, notably IL-8, IL-6, IFN **γ**, VEGF and COMP (*r* values = 0.22–0.33, *P* < 0.014–0.0001) and with CRP and SAA levels (*r* = 0.40 and 0.41, resp., *P* < 0.0000) and SDAI (*r* = 0.29, *P* < 0.0001), but not with erosions or nodulosis. However, the correlations of CRP and SAA with SDAI were stronger (respective values of 0.63 and 0.54, *P* < 0.001 for both). COMP correlated with smoking, RF, and MMP-3. MMP-3 is significantly associated with disease activity, inflammatory mediators and cartilage breakdown, making it a potential biomarker of disease severity, but seemingly less useful than CRP and SAA as a biomarker of disease activity in early RA.

## 1. Introduction

Rheumatoid arthritis (RA) is characterised by a progressive, erosive polyarthritis,resulting in immune-mediated joint destruction and eventual disability.Components of the immunoinflammatory response include acute phase proteins, auto-reactive T cells, B cells, and their respective inflammatory mediators. The consequence is a self-perpetuating chronic inflammatory response involving a complex interplay between infiltrating inflammatory cells and the structural cells of the synovial joint [1, 2]. 

 Matrix metalloproteinase-3 (MMP-3) is produced predominantly by chondrocytes and synovial fibroblasts and is found in high concentrations in the synovial fluid, with elevated levels also found in the serum of RA patients [3]. MMP-3 production is upregulated by the proinflammatory cytokines IL-1*β*, TNF, IFN *γ*, and IL-17A, as well as by serum amyloid A (SAA), an acute phase reactant, with counter-regulatory inhibition from IL-4 and IL-13 [4–10]. Cytokine/SAA-driven production of MMP-3 in the rheumatoid joint appears to be a key mediator of cartilage destruction, while bone resorption is facilitated by MMP-3-mediated removal of the outer osteoid layer, enabling attachment of osteoclasts to the underlying bone [11]. Moreover, MMP-3 mediates the proteolytic activation of pro-MMP-9 released from both inflammatory and structural cells [4]. Aside from its role in promoting the recruitment of neutrophils, monocytes, T cells, and osteoclasts [4], MMP-9 promotes the release of membrane-bound vascular endothelial growth factor (VEGF), thereby contributing to angiogenesis and disease progression [12]. 

With respect to its diagnostic and prognostic potential, elevated serum concentrations of MMP-3, while having no specificity for RA, have been found in many [13–21], but not all [22–25], studies to correlate with disease severity and response to chemotherapy. However, the global interrelationships of MMP-3 with proven genetic markers of disease susceptibility, such as the shared epitope (SE), as well as with other systemic biomarkers such as autoantibodies, cytokines, cartilage breakdown products, and the acute phase reactants CRP and SAA in particular, have not been well characterised. 

CRP and SAA are synthesised in the liver in response to proinflammatory cytokines such as TNF, IL-1, and IL-6. Monocytes/macrophages, endothelial cells, synovial fibroblasts, and chondrocytes can also produce SAA. Unlike CRP, SAA is also locally expressed and accumulates in inflamed tissue with histological studies demonstrating expression of SAA in the perivascular and lining layer of RA synovial tissue, in regions of leukocyte recruitment and angiogenesis, especially at the cartilage pannus junction. SAA is apparently a more sensitive marker of inflammation in RA than CRP, with the SAA/CRP ratio, possibly being of more significance [26]. SAA augments the inflammatory response in a cytokine-like fashion by attracting monocytes/macrophages, leukocytes and T lymphocytes, while promoting neutrophil survival and endothelial activation and stimulating the production of the proinflammatory mediators TNF, IL-1, IL-6, IL-8, and IL-17, thus initiating an amplifying loop. Exposure of synovial fibroblasts and chondrocytes to SAA promotes MMP-3 mediated adhesion molecule expression, as well as phagocytosis and chemotaxis of monocytes and neutrophils, thereby contributing to synovial inflammation, hyperplasia, angiogenesis, and joint destruction [9]. SAA has also been implicated in the pathogenesis of atherosclerosis and premature cardiovascular disease, an important aspect in the management of patients with RA, thus making it a potential target for therapy to control both RA and its complications. 

The objectives of the current study were to assess the relationships between circulating MMP-3 and (i) SE; (ii) biomarkers of inflammation and cartilage degradation; (iii) disease activity and radiographic changes in comparison with CRP and SAA in a cohort of predominantly black female South Africans with early disease-modifying antirheumatic drugs (DMARD) naive RA. 

## 2. Patients and Methods

### 2.1. Patients

Baseline data was reviewed from 128 patients who were part of the Gauteng Rheumatoid Arthritis Trial (GREAT), a prospective study of early DMARD naive RA patients [27–29]. Patients were recruited from the rheumatology clinics of two tertiary hospitals, Chris Hani Baragwanath Hospital and Steve Biko Academic Hospital, attached to the Universities of the Witwatersrand and Pretoria, South Africa, respectively, and the study was approved by the Research Ethics Committees of the Faculties of Health Sciences of both institutions. 

Demographic data, duration of symptoms, education level, medication history, clinical assessment including 28 joint count, and the presence of extra-articular involvement were recorded, as was disease activity measured according to the Health Questionnaire Disability Index (HAQ-DI) and Simplified Disease Activity Index (SDAI), and have been described in detail elsewhere for this cohort of patients [27–29]. 

Radiographs of the hands and feet were scored according to the modified Larsen score. In each case, 32 joint areas were scored: 8 PIP (proximal interphalangeal), 2 thumb IP (interphalangeal), 10 MCP (metacarpophalangeal), 4 areas in each wrist, and 8 MTP (metatarsophalangeal) joints. The maximum score that could be attained was 160. Radiographs were also assessed for presence or absence of erosive disease. 

### 2.2. Laboratory Methods

Venous blood was collected as described previously, followed by prompt separation of serum and storage at minus 20°C until analysed. 

#### 2.2.1. Autoantibodies, Acute Phase Reactants, and Cytokines/Chemokines

These were assayed using nephelometric, immunofluorimetric, and multiplex suspension bead array procedures as previously described [27], while haemoglobin concentrations and erythrocyte sedimentation rates were measured using standard haematological procedures. 

#### 2.2.2. MMP-3

Serum concentrations of MMP-3 were measured using the Quantikine Total MMP-3 Immunoassay (ELISA) according to the instructions supplied by the manufacturer (R & D Systems, Minneapolis, MN, USA) and the results expressed as nanograms (ng)/mL serum. Serum levels were measured in healthy controls (15 females and 10 males, age range 24–64). 

#### 2.2.3. Cartilage Oligomeric Matrix Protein (COMP)

Serum levels were measured using the Kamiya Human COMP ELISA system (Kamiya Biomedical Company, Seattle, WA, USA) and the results expressed as micrograms/mL. Serum levels were also measured in 8 of the previous control subjects. 

#### 2.2.4. Shared Epitope (SE) Genotyping

This was performed as described previously using high-resolution reverse sequence-specific oligonucleotide probes and Luminex technology [27], with classification as SE or non-SE genotypes according to the method of Tezenas Du Montcel et al. [30, 31]. 

#### 2.2.5. Statistical Methods

Descriptive statistics of continuous variables were done by using a measure of central tendency (mean or median) and a measure of variability (standard deviation or range). Frequencies and percentages were used to describe categorical variables of the nominal or ordinal scales. Cytokine data were not normally distributed, and therefore the Spearman's pairwise correlations were used to find the strength of each cytokine, demographics with MMP-3 and COMP, and between themselves. A *P* value of <0.05 was considered statistically significant, while, in the case of the cytokines, a *P* value of <0.003 following correction for multiple comparisons was used. Statistical analysis was done using STATA version 12.0 (Stata Corporation, College Station, TX, USA). 

## 3. Results

### 3.1. Demographic, Clinical, and Laboratory Data (Autoantibodies, Acute Phase Reactants, Haemoglobin, ESR, MMP-3, and COMP)

As seen in Table 1, the majority of patients were middle-aged black females, with a mean disease duration of almost 12 months. Patients had high disease activity as reflected by a mean DAS of 6.28 and moderate to high functional disability. Also, 82% of patients were RF positive and 81% ACPA positive. Erosions were seen in 52%, and the mean Larsen score was 22.4 (±12.7).

 Serum MMP-3 levels were significantly (*P* < 0.0001) higher in the RA patients relative to the healthy control subjects. Using cut-off values of 18 ng/mL and 1.99 *μ*g/mL for MMP-3 and COMP, respectively (based on the mean values for the healthy control subjects +2 standard deviations), the mean MMP-3 and COMP values for the control groups compared favourably with previous studies of 46 and 291 healthy controls respectively [32, 33]. The respective frequencies of elevated MMP-3 and COMP levels for the RA patients were 56.25% and 34.6% (Table 1). 

### 3.2. Correlations of MMP-3 with Clinical Indices of Disease Activity and Noncytokine, Inflammatory Biomarkers

Correlations that achieved statistical significance are shown in Table 2, which demonstrates associations of serum MMP-3 with SDAI, CRP, SAA, RF and COMP. There were no statistically significant associations of MMP-3 with HAQ-DI, tender joint count, nodules, erosions, Larsen score or ACPA. 

### 3.3. Correlations of MMP-3 with Cytokines/Chemokines/Growth Factors

We have previously reported that the circulating cytokine/chemokine/growth factor concentrations in this cohort of RA patients are significantly higher than in healthy control subjects [27]. In the current study, circulating MMP-3 was found to correlate positively and significantly with IL-6, IL-8, IL-12, IFN-*γ*, and VEGF (*r* values 0.27–0.33, *P *values <0.003–0.0001), the strongest correlations being observed with IL-8 > IL-6> IFN-*γ* and VEGF (Table 3). 

### 3.4. Correlations of COMP with Clinical Indices of Disease Activity and Noncytokine Inflammatory Biomarkers

COMP correlated positively and significantly with RF (*r* = 0.23, *P* < 0.006), but not with any of the clinical indices or other inflammatory biomarkers. A weak positive correlation between COMP and smoking history (ever smoked) was also noted (*r* = 0.17, *P* < 0.04). 

### 3.5. Association of MMP-3, SAA, CRP and COMP with SE

Subgroup analysis revealed no differences in MMP-3, SAA, CRP, or COMP levels in risk allele negative or positive patients. 

### 3.6. Correlations of CRP and SAA with Clinical and Laboratory Indices of Disease Activity

Given the key role of SAA in activating the synthesis of MMP-3 by synovial chondrocytes and fibroblasts [9, 34], correlations of this acute phase reactant, as well as those of CRP, with clinical and traditional noncytokine indices of disease activity were determined and these are shown in Table 4. Correlations included evaluation of the SAA/CRP ratio, but this did not reveal any significant associations (data not shown). Both acute phase proteins, especially CRP, were found to have stronger associations with disease activity in early RA than MMP-3. 

## 4. Discussion

Consistent with data from other studies finding elevated MMP-3 levels in 62%–80% of patients [14, 35], MMP-3 levels were found to be elevated in 56.25% of our patients. Although no correlations with SE were detected, MMP-3 was found to correlate significantly with measures of disease activity, specifically SDAI, ESR, CRP, and SAA. In addition to these, correlations with predominantly proinflammatory cytokines, especially IL-8, IL-6, IFN*γ*, and VEGF (Table 3), were also observed. Visvanathan et al. in a study of 144 patients with RA showed significant correlations with IL-8, IL-1*β* and CRP, the strongest correlation being with CRP (*r* = 0.601, *P* < 0.001) [36]. A smaller study by Ribbens et al. in 20 patients with RA showed correlations of MMP-3 and disease activity score (DAS), CRP, and IL-6 level [20]. 

 Few studies have examined serum levels of MMP-3 in a DMARD naïve early RA cohort of predominantly black females, exploring the associations with clinical parameters of disease activity, acute phase response, SE, and a wide range of cytokines, chemokines, and growth factors. 

Recent developments in the management of RA are aimed at rapid disease control, as well as early introduction of efficient therapies, which makes the search for useful biomarkers such as MMP-3 important. This contention is underscored by an interest in the development of an automated serum detection procedure for MMP-3 and the recent introduction of a commercially available multibiomarker disease activity (MBDA) test. The MBDA test includes MMP-3 in the quantitative assessment of disease activity, having shown significant correlations with DAS 28-CRP (AUROC = 0.77; *P* < 0.001 for seropositive patients and AUROC = 0.70; *P* < 0.001 for seronegative patients). 

In the current study, the levels of circulating SAA, a mediator of synthesis of MMP-3, as well as those of CRP, were found to be elevated in 74.4% and 76.1% of patients, respectively, and correlated significantly with measures of disease activity, as described in previous studies, some of which suggest that SAA is a more sensitive marker in RA than CRP. However, this was not confirmed in the current study, with CRP being equivalent or slightly superior to SAA as a marker of disease activity in early RA. Because SAA is also locally released and expressed in the synovial tissue, we reasoned that the SAA/CRP ratio may be a more accurate index of disease activity than the individual biomarkers [26]. However, this did not prove to be the case in our cohort. With regard to poor prognostic markers, such as the presence of nodules, erosive disease, and the Larsen score, correlations with CRP and SAA, but not with MMP-3, were evident. No associations of SAA or CRP with the SE risk alleles or COMP were observed. 

Importantly, the correlations of both CRP and SAA with SDAI were considerably stronger than those of MMP-3 with SDAI, demonstrating that measurement of either of these acute phase reactants, but preferably CRP, is probably the best serological determinant of disease activity in early RA. 

Cartilage destruction is one of the consequences of uncontrolled synovial proliferation, with serum levels of cartilage degradation products such as COMP having been shown to be associated with RA disease activity and future radiological damage [37]. In the current study, COMP levels were elevated in 34.6% of patients compared to other studies showing percentage of elevated COMP levels ranging from 41% to 67% [38, 39]. COMP was found to correlate weakly with smoking history (ever smoked), RF, and MMP-3, but no correlations were found with measures of disease activity as reflected in some previous studies which described correlations with CRP, DAS, ESR, and rheumatoid nodules [40]. MMP-3 is an important effector of cartilage metabolism; hence, the correlation with COMP is not surprising. Although no correlations between COMP and radiographic changes were found, this may simply reflect increased cartilage turnover rather than destruction in early disease [40]. 

The correlation of MMP-3 with COMP and lack thereof with the acute phase reactants studied may suggest that MMP-3 is a better biomarker to predict disease progression; thus, combining measurement of CRP with that of MMP-3 may be a useful strategy predict disease progression [15]. 

In conclusion, the most significant and original findings of the current study are (i) elevated levels of circulating MMP-3, which may identify a subset of RA patients likely to develop severe disease, are significantly associated with SDAI, proinflammatory cytokines, and most strongly with CRP and SAA; (ii) CRP and SAA are more strongly correlated with disease activity in early RA than MMP-3; (iii) measurement of either CRP or SAA in combination with MMP-3 on presentation may be useful in the assessment of disease activity and prediction of progression in early RA. 

## Figures and Tables

**Table 1:** Demographic, clinical, and laboratory data for the group of RA patients (*n* = 128).

Parameters	Mean	SD
Age (years)	46.5	12.79
Female (%)	102 (79.6)	
Ethnic origin: Black %	119 (92.97)	
DAS ESR	6.28	1.17
Smoking (%)	28 (21.8)	
Nodule (%)	30 (23.4)	
Erosions (%)	64 (52.03)	
mHAQ-D1	1.72	0.76
Disease duration	11.70	7.11
SJC	3.51	4.08
ESR	45.02	27.64
RF (IU/mL)	515.83	522.099
aCCP (U/mL)	679.74	626.54
CRP (*µ*g/mL)	26.95	33.95
SAA (ug/mL)	66.00	128.54
Hb (g/L)	12.8	1.76
MMP-3: control group (ng/mL)	7.5	5.4
MMP-3 (% elevated)	35.8 (56.3)	43.9
Comp: control group (*µ*g/mL)	1.11	0.44
Comp (% elevated)	1.85 (92.0)	0.77

DAS: disease activity score.

HDA: high disease activity.

SJC: swollen joint count.

RF: rheumatoid factor.

aCCP: anticyclic citrullinated peptide antibodies.

CRP: C-reactive protein.

SAA: serum amyloid A.

Hb: haemoglobin.

ESR: erythrocyte sedimentation rate.

MMP: matrix metalloproteinase.

Comp: cartilage oligomeric protein.

**Table 2:** Correlations of MMP-3 with indices of disease activity and noncytokine biomarkers.

Parameter	*r*	P*	*n*
SDAI	0.29	0.0009	128
CRP	0.39	0.0000	115
SAA	0.40	0.0000	115
COMP	0.21	0.0148	127
RF	0.19	0.0320	128

*P level significance < 0.05.

**Table 3:** Correlations of MMP-3 with cytokines.

Cytokine	*r*	P*	*n*
IL-8	0.33	0.0001	128
IL-6	0.30	0.0005	128
IFN*γ*	0.28	0.0010	128
VEGF	0.28	0.0014	128
IL-12	0.27	0.0024	128

*P level of significance of <0.003.

**Table 4:** Correlations of SAA and CRP with clinical and laboratory indices of disease activity.

Parameter	SAA*	CRP*
	0.25	0.35
SJC	0.007	0.0001
	115	115
	0.29	0.39
DAS ESR	0.002	0.0000
	115	115
	0.54	0.63
SDAI	0.0000	0.0000
	115	115
	0.18	0.22
NODULE	0.047	0.014
	115	115
	0.83	—
CRP	0.0000	—
	115	—
	0.24	0.25
RF	0.009	0.006
	115	115
	0.21	NS
aCCP	0.023	—
	115	—
	0.36	0.38
Erythrocyte sedimentation rate	0.0001	0.0001
	115	115
	−0.34	−0.26
Haemoglobin	0.0002	0.005
	110	110
	0.26	0.24
Platelets	0.005	0.01
	110	110
	0.21	0.19
Erosion	0.023	0.04
	110	110
	0.20	0.32
Larsen score	0.044	0.0006
	106	106
	NS	0.28
Tender joint count	—	0.002
	—	115

*For each pair of values, the correlation coefficients are uppermost with the corresponding *P* and *n* values underneath.
